# Academy of Sciences—Prizes

**Published:** 1864-04

**Authors:** 


*Medical and Surgical Reporter.*

---

ACADEMY OF SCIENCES—PRIZES. The Academy of Sciences lately held its annual session at the Institute, Dr. Velpeau presiding. The prize for *Experimental Physiology* was given to Dr. Moreno. For *Meaicine and Surgery*, the Academy awarded to Dr. Chassaignac a prize of 2,500 francs, and to Drs. Bourdon, Cahen, Debont, and Gallois, an honorable mention with 1,500 francs for each. For *Insalubrious Arts* a prize of 2,500 francs was given to M. Grimaux de Caux, for his work, "On the supply of Water to Towns and Rural Habitations." A prize of 2,500 francs to M. Guignet for the preparation of non-injurious green or printing on tissues; and a recompense of 1,500 francs to M. Bouffe for having discovered a substitute for arsenical green in the manufacture of artificial flowers.

The following are the prizes on medical subjects proposed for this year and the ensuing ones by the Academy, the papers in all cases to be sent in headed with some motto, to be repeated on a sealed envelope containing the name of the author. 5,000 fr. "To give a complete history of Pellagra," (March 31st, 1864). 5,000 fr. "On the application of electricity to therapeutics," (March 31st, 1866). 20,000 fr., the Academy and Emperor's prize, "For method of preserving members by preserving the periosteum," (March 31st, 1866). Lastly, the *Breant* prize of 100,000 fr. for the discovery of an unquestionable specific against cholera, or, in default of this, a prize of 4,000 fr. to the competitor who can prove that there exist in the air substances which may materially contribute to the propagation of epidemical diseases.

